# Prevalence of metabolic syndrome in adolescents based on three diagnostic definitions: a cross-sectional study

**DOI:** 10.20945/2359-3997000000634

**Published:** 2023-05-29

**Authors:** Dejane de Almeida Melo, Alcione Miranda dos Santos, Victor Nogueira da Cruz Silveira, Michele Bezerra Silva, Alcides da Silva Diniz

**Affiliations:** 1 Universidade Federal de Pernambuco Programa de Pós-graduação em Nutrição Recife PE Brasil Programa de Pós-graduação em Nutrição, Universidade Federal de Pernambuco, Recife, PE, Brasil; 2 Universidade Federal do Maranhão Departamento de Saúde Pública São Luís MA Brasil Departamento de Saúde Pública, Universidade Federal do Maranhão, São Luís, MA, Brasil; 3 Universidade Federal do Maranhão Programa de Pós-graduação em Saúde Coletiva São Luís MA Brasil Programa de Pós-graduação em Saúde Coletiva, Universidade Federal do Maranhão, São Luís, MA, Brasil; 4 Universidade Federal de Pernambuco Departamento de Nutrição Recife PE Brasil Departamento de Nutrição, Universidade Federal de Pernambuco, Recife, PE, Brasil

**Keywords:** Metabolic syndrome, adolescents, cardiometabolic risk factors

## Abstract

**Objective:**

There is no consensus as to the best criterion for the evaluation of metabolic syndrome (MS), impairing the estimation of its prevalence. This study aims to compare MS estimates using three recommended definitions for adolescents based on a cross-sectional study nested in the Consortium of Brazilian Birth Cohorts in São Luís, Maranhão.

**Subjects and methods:**

A total of 2,515 adolescents aged between 18 and 19 years were evaluated. The criteria of International Diabetes Federation (IDF) and National Cholesterol Education Program Panel III (NCEP-ATP) modified by Cook and cols. (2003) and De Ferranti and cols. (2004) defined SM. To compare the estimates of MS prevalence, the chi-square, Fisher’s exact and Cohen’s Kappa index tests were used.

**Results:**

Among the 2,064 participants evaluated in the final sample. The prevalence of MS ranged from 4.2% (95% CI: 3.3-5.1) to 10.2% (95% CI: 8.8-11.4). When comparing the estimates of MS prevalence in the total sample and by sex, a statistically significant difference was observed. The agreement between the criteria ranged from 0.42 (CI 95%: 0.35-0.49) to 0.55 (CI 95%: 0.48-0.62) in the total sample, 0.33 (CI 95%: 0.24-0.42) to 0.59 (95%CI: 0.47-0.71) among boys and 0.39 (95% CI: 0.26-0.52) to 0.54 (95% CI: 0.44-0.64) among girls.

**Conclusion:**

Different criteria provide different estimates for the prevalence of MS in adolescents, reflecting the importance of establishing a consensus.

## INTRODUCTION

In 1988, Gerald M. Reaven proposed the existence of a series of closely related variables which tended to occur in the same individual. He called it “syndrome X”, which came to represent enormous importance in the genesis of cardiovascular disease atherosclerosis (1).

Subsequently, metabolic syndrome (MS) was defined as an aggregate of clinical conditions that comprise central obesity, systemic arterial hypertension, insulin resistance (or type 2 diabetes mellitus), and atherogenic dyslipidemia (2). It is a construct used to identify individuals at a higher risk for cardiovascular events (3).

With the advancement of research involving MS, several classifications have been proposed to identify MS in adolescents. More than 40 definitions for MS diagnosis in this age group have already been reported (4).

In this context, the term MS became permeated by controversies reported in the literature due to the various criteria used for its definition, which remains without a consensus so far (5). As a result, it affects the estimation of its prevalence, the measurement of outcomes, and the comparability between studies (6). In addition, there are still important gaps regarding the procedures that should guide the assessment of adolescents in the transition to adulthood. In adults, the criteria for defining MS are not based on the percentile distribution commonly used in the pediatric age group but on fixed values (7).

The development of MS in early adulthood can lead to a high-risk burden for cardiovascular disease throughout life (8), with repercussions 25 to 30 years after exposure to MS in childhood (9). Therefore, there is a need for a more accurate assessment of this condition.

We hypothesize that different definitions may result in different estimates of prevalence for the same event. This study aimed to compare the estimates of MS prevalence, using three definitions recommended for its screening, in adolescents in a population-based sample from the RPS (Ribeirão Preto, Pelotas, São Luís) Consortium of Brazilian birth cohorts in the city of São Luís (MA).

## SUBJECTS AND METHODS

### Design and setting

This study is cross-sectional and nested in a birth cohort. This study was carried out in the city of São Luís – MA, northeastern Brazil, which comprises the RPS Consortium of Brazilian birth cohorts. Studies from Brazilian cohorts aim to investigate the early determinants of health in childhood, adolescence, and adulthood and collect data on nutritional and health status (10).

The study was conducted in ten public and private hospitals in the city from March 1997 to February 1998. It comprised the beginning of the cohort at the time of its members’ birth. The population-based birth sample in São Luís corresponded to 96.3% of births in the study period, excluding non-hospital births and those in hospitals with less than 100 births per year. Systematic sampling was used with proportional stratification according to the number of births in each maternity hospital, one in seven deliveries. A total of 2,831 births were obtained. Excluding non-residents in São Luís, twins, and stillbirths, the final sample included 2,443 births, with 5.8% of losses due to refusals or early discharge. This cohort was followed at 7-9 years and again at 18-19 years (11). Simões and cols. (11) provide more details about the methodology used to select the participants (11).

This study considered data from participants in the third phase of the RPS Cohort in São Luís (MA). The third phase of this cohort took place in 2016, with participants aged between 18 and 19. Data collection took place on the premises of the Federal University of Maranhão (UFMA). Health professionals were hired, who were trained to apply the survey questionnaires and/or handle the equipment.

The sample size calculation was based on the results of 50 adolescents randomly selected from the database under study, whose prevalence of MS was 4%. Considering the size of the eligible population and aiming at more accurate estimates, we used a 1.5% margin of error for the sample size calculation. Thus, the minimum sample required to identify MS comprised 495 individuals.

The RPS Cohort – São Luís (MA) sample included 2,515 adolescents. However, in this study, 451 adolescents were excluded because they had missing and/or inconsistent information regarding the variables of interest. Thus, the final sample included 2,064 participants.

### Study variables

Sociodemographic and economic variables were evaluated; age (in years), sex, education (in degrees of education), self-reported color, and social class (12).

To assess life habits, the variables “current smoking”, “past smoking” habits, and “alcohol consumption” pattern were analyzed. The alcohol consumption pattern was assessed using the Alcohol Use Disorder Identification Test (AUDIT) instrument, categorized as low/abstinence, risk, harmful, and probably dependent (13). The “past smoking” habit variable was obtained from the answer (yes/no) to the question: “Have you ever smoked cigarettes at least once a week”. The “current smoking” variable was obtained from the question: “Do you still smoke cigarettes?”.

The anthropometric data evaluated were weight, height, and waist circumference. All anthropometric measurements were performed following guidelines for the collecting and analyzing of anthropometric data in health services available in the Technical Standard of the Food and Nutrition Surveillance System (14). Each participant’s weight (in kilograms) was measured on a high-precision digital scale connected to the Gold Standard BOD POD equipment (COSMED Metabolic Company^®^, Rome, Italy). Height (in centimeters) was measured with the Altura Exata stadiometer (Trident Indústria de Precisão Ltda.^®^, Brazil). Body mass index (BMI) was obtained *a posteriori* through the ratio between weight in kg and height in square meters. Waist circumference (WC) was measured in centimeters using the Photonic Scanner ([TC] 2 Labs^®^, USA), which makes a three-dimensional image of the human body and obtains measurements of different body circumferences.

Systolic blood pressure (SBP) and diastolic blood pressure ­(DBP) were calculated based on the mean of the three measurements taken on the Omron HEM 742INT device (Omron^®^, São Paulo, Brazil), obtained after at least five minutes at rest.

For biochemical analysis, 40 mL blood samples were obtained from the cubital vein aseptically by an experienced technician. Fasting was not required. Random plasma glucose (an assessment that does not consider what was consumed from the last meal), high-density lipoprotein (HDL), and triglycerides (TG) were analyzed by the automated enzymatic colorimetric method using the Roche^®^ Cobas c501 equipment.

The person responsible for the collection centrifuged the material and stored the serum and part of the whole blood in a freezer at -20 °C. The rest of the sample was taken to the Clinical Analysis Laboratory of the University Hospital of the Federal University of Maranhão, and the test results were entered into RedCap. In addition, a fellow took frozen material weekly to the Genetics Laboratory (Labgen) for cataloging and storage in freezers at -80 °C.

According to Bowen and cols. (15), plasma glucose represents an important indicator of dysglycemia, which may play an important role in tracking and identification strategies. In turn, Lee and cols. (16) observed that the assessment of non-fasting serum glucose levels has the potential to be incorporated into clinical practice as an initial screening and to determine which individuals should undergo definitive tests.

The Update of the Brazilian Directive on Dyslipidemias and Prevention of Atherosclerosis (17) recommends that fasting is not necessary to perform CT and HDL, as the postprandial state does not interfere with the concentration of these particles. Moreover, increased postprandial TG values represent a higher risk for cardiovascular events.

Since there are more than 40 criteria reported in the literature for the definition of MS (4), we consulted those cited by the Department of Nutrology of the Brazilian Society of Pediatrics in its Guidance Manual for Obesity in Childhood and Adolescence (18) and in the Guidelines of the Brazilian Society of Diabetes (7). Furthermore, considering the age of the sample under study and the variables available for analysis, MS was assessed using three definitions; International Diabetes Federation (IDF) (19) and National Cholesterol Education Program Panel III (NCEP-ATP) modified by Cook and cols. (20) and De Ferranti and cols. (21) (Table 1).

**Table 1:** Definitions for assessing metabolic syndrome in Adolescents

Criterion	Definition	WC	BP	TG	HDL	Glycemia
IDF (2007)	High WC + 2 or more criteria	≥94^a^ and ≥80^b^	≥130/85^e^	≥150^e^	<40^a^ and <50^b^	≥100^f^
Cook and cols. (2003)	3 or more criteria	≥p 90^c^	≥p 90^d^	≥110	≤40	≥110
De Ferranti and cols. (2004)	3 or more criteria	>p 75^c^	≥p 90^d^	≥100	<50^a^ and <45^b^	≥110

a Boys;

b girls;

c for age and sex;

d for age, sex and height

e or pharmacological treatment,

f or diagnosis of diabetes.

WC: waist circumference in cm; BP: blood pressure in millimeters of mercury; TG: triglycerides in milligrams per deciliter; HDL: high-density lipoprotein in milligrams per deciliter; Glycemia: fasting and in milligrams per deciliter.

In addition to assessing the presence of MS (yes/no), adolescents were stratified according to the number of unfavorable components: 0 - no component, 1 - one component, 2 - two components, and 3 - three or more components.

Data collection took place at the Federal University of Maranhão (UFMA). Health professionals were hired and trained to apply the survey questionnaires and/or handle the equipment.

The project “Determinants throughout the Life Cycle of Obesity, Precursors of Chronic Diseases, Human Capital and Mental Health: A Contribution of the São Luís Birth Cohorts to the SUS” was approved by the Research Ethics Committee of the University Hospital of the Federal University do Maranhão through the process no. 1,302,489. All participants signed the Free and Informed Consent Form. This project was carried out following the Declaration of Helsinki and the requirements of Resolution 466/12 of the National Health Council and its complementary ones. Moreover, the data analysis referring to adolescents evaluated by the RPS Consortium in São Luís (MA) was approved by the Ethics and Research Committee of the Federal University of Pernambuco – Academic Center of Vitória (CAEE: 48597421.8.0000.9430).

### Statistical analysis

The R Studio software (R Core Team^®^ version 4.0.5) was used for the statistical analysis of the data.

Initially, an exploratory analysis of the data was carried out to exclude outliers. Then, a descriptive analysis of the data was carried out. Finally, the categorical variables were presented through frequencies and percentages. The variability was presented by approximating the binomial distribution to the normal distribution by a 95% confidence interval.

The Chi-Square and Fisher’s Exact tests were used to assess the differences between the proportions. The agreement between the different definitions was assessed using Cohen’s Kappa index, whose valuation was defined according to that proposed by Laddis & Koch (22). The percentiles for the WC and BP variables were defined according to the population under analysis, specific for sex, age, and height when necessary. The significance level was set at 5%.

## RESULTS

Among the 2,064 participants evaluated, 50.8% (95% CI: 48.6-53.0) were female and mostly aged 18 years (75.0% 95% CI: 73.1-76.9). Most of the adolescents evaluated attended high school (60.8% 95% CI: 58.2%-62.9%), were of self-declared brown skin color (63.4% 95% CI: 61.2%-65.4%), and belonged to social class C (44.9% 95% CI: 42.8%-47.1%) (data not shown in the table).

As for life habits, most adolescents had a low-risk pattern for alcohol consumption (81.0% 95% CI: 79.2%-82.6%). In terms of smoking, 91.2% (95% CI: 89.9%-92.3%) reported not having smoked in the past, and only 5.1% (95% CI: 4.1%-6.2%) reported currently smoking (data not shown in table).

According to the criteria used to estimate the prevalence of MS in the study sample, we obtained the following estimates: IDF (19) – 4.9% (95% CI: 4.0-5.9), Cook and cols. (20) – 4.7% (95% CI: 3.9-5.6) and De Ferranti and cols. (21) – 11.2% (95% CI: 9.9-12.7). When assessing MS by sex, female adolescents had a higher prevalence according to the IDF (19) criteria (*p* = 0.017). However, when MS was assessed using the criteria of Cook and cols. (20) and De Ferranti and cols. (21), the prevalence of MS was higher in males (*p* < 0.05) (Table 2).

**Table 2:** Prevalence of Metabolic Syndrome in adolescents aged between 18 and 19 years of the RPS Birth Cohorts. São Luís, 2016

Definition	Total (n = 2,064)				Boys (n = 1,015)				Girls (n = 1,049)			*p*-value
	n	%	95%CI		n	%	95%CI		n	%	95%CI	
IDF (2007)	101	4.9	4.0-5.9		38	3.7	2.6-5.1		63	6.0	4.6-7.6	0.017
Cook and cols (2003)	98	4.7	3.9-5.6		64	6.3	4.9-8.0		34	2.8	2.3-4.5	0.001
De Ferranti and cols (2004)	232	11.2	9.9-12.7		140	13.8	11.7-16.1		92	8.8	7.2-10.7	<0.001

IDF: International Diabetes Federation; 95% CI: 95% confidence interval.

When evaluating the frequency of the number of MS components in the total sample (Table 3), the range of 2 and 3 or more components obtained lower frequencies. This trend was also observed when the sample was stratified by sex.

**Table 3:** Prevalence of the quantitative components of the Metabolic Syndrome by different criteria in adolescents aged 18 and 19 years of the RPS Birth Cohorts. São Luís, 2016 (n = 2,064)

Criteria	Scores	Total (n = 2,064)				Boys (n = 1,015)				Girls (n = 1,049)			*p*-value
		n	%	95% CI		n	%	95% CI		n	%	95% CI	
IDF (2007)	0	799	38.7	36.6-40.8		483	47.6	44.4-50.7		316	30.1	27.3-33.0	<0.001
	1	737	35.7	33.6-37.8		341	33.6	30.7-36.6		396	37.8	34.8-40.7	
	2	413	20.0	18.3-21.8		142	14.0	11.9-16.3		271	25.8	23.2-28.6	
	≥3	115	5.6	4.6-6.6		49	4.8	3.6-6.4		63	6.3	4.6-7.6	
Cook and cols. (2003)	0	1107	53.6	51.4-55.8		453	44.6	41.5-47.7		654	62.3	59.3-65.2	<0.001
	1	622	30.1	28.2-32.1		337	33.2	30.3-36.2		285	27.2	24.5-29.9	
	2	237	11.5	10.1-12.9		161	15.9	13.6-18.2		76	7.2	5.0-9.0	
	≥3	98	4.8	3.9-5.6		64	6.3	4.9-8.0		34	3.2	2.3-4.5	
De Ferranti and cols. (2004)	0	667	32.3	30.3-34.4		291	28.7	25.9-31.5		376	35.8	32.9-38.8	<0.001
	1	751	36.4	34.3-38.5		367	36.2	33.2-39.2		384	36.6	33.6-39.6	
	2	414	20.1	18.3-21.8		217	21.4	18.9-24.0		197	18.8	16.4-21.3	
	≥3	232	11.2	9.9-12.7		140	13.8	11.7-16.1		92	8.8	7.2-10.7	

95% CI: 95% confidence interval; IDF: International diabetes Federation.

Table 4 presents the frequencies from each MS component. The definitions of the IDF (19) and De Ferranti and cols. (21) identified higher frequencies of high WC compared to the criterion by Cook and cols. (20) in the total sample. Regarding blood glucose, we found a higher frequency of deviations by the IDF (19) definition than the others. Elevated TG and reduced HDL showed higher prevalence considering the definition by De Ferranti and cols. (21).

**Table 4:** Prevalence of the components of the Metabolic Syndrome in adolescents aged 18 and 19 years of the RPS Birth Cohorts. São Luís, 2016

Criteria	Components	Total (n=2,064)				Boys (n=1,015)				Girls (n=1,049)			*p*-value
		N	%	95%CI		n	%	95%CI		n	%	95%CI	
IDF (2007)	WC	579	28.1	26.1-30.0		118	11.6	9.7-13.7		461	43.9	40.9-47.0	<0.001
	Blood glucose	393	19.0	17.3-20.8		211	20.8	18.3-23.4		182	17.3	15.1-19.8	0.053
	TG	174	8.4	7.7-9.7		113	11.1	9.2-13.2		61	5.8	4.5-7.4	<0.001
	HDL	726	35.0	33.1-37.2		293	28.9	26.1-31.7		433	41.3	38.2-44.3	<0.001
	BP	59	2.8	2.2-3.6		49	4.8	3.6-6.4		10	1.0	0.4-1.8	<0.001
Cook and cols. (2003)	WC	206	10.0	8.7-11.3		106	10.4	8.6-12.5		108	10.3	8.5-12.3	0.917
	Blood glucose	185	9.0	7.7-10.2		99	9.8	8.0-11.8		86	8.2	6.6-10.0	0.216
	TG	473	22.9	21.1-24.8		296	29.2	26.4-32.0		177	16.9	14.6-19.3	<0.001
	HDL	459	22.2	20.4-24.1		320	31.5	28.6-34.5		139	13.3	11.3-15.4	<0.001
	BP	81	3.9	3.1-4.8		41	4.0	2.9-5.5		40	3.8	2.7-5.2	0.791
De Ferranti and cols. (2004)	WC	509	24.7	22.8-26.5		255	25.1	22.5-27.9		26.4	25.2	22.6-27.9	0.982
	Blood glucose	185	9.0	7.7-10.2		99	9.8	8.0-11.8		86	8.2	6.6-10.0	0.216
	TG	612	29.7	27.6-31.6		367	36.2	33.2-39.2		245	23.4	20.8-26.0	<0.001
	HDL	918	44.5	42.3-46.6		485	47.8	44.6-50.9		433	41.3	38.2-44.3	0.002
	BP	81	3.9	3.1-4.8		41	4.0	2.9-5.5		40	3.8	2.7-5.2	0.791

95% CI: 95% confidence interval; IDF: International diabetes Federation; WC: waist circumference; HDL: high-density lipoprotein; TG: triglycerides; BP: blood pressure. *High/low according to each criterion and cut-off points defined in Table 2.

When stratified by sex, there was a statistically significant difference (*p* < 0.05) for all MS criteria evaluated according to the definition of the IDF (19) (except blood glucose). Girls had higher frequencies of high WC and reduced HDL, and boys had higher frequencies of TG and high BP. According to the definitions by Cook and cols. (20) and De Ferranti and cols. (21), there was a statistically significant difference only for TG and HDL, and the frequency of deviations in these variables was higher in males (Table 4).

Table 5 shows the agreement between the different definitions evaluated. In the total sample, there was moderate agreement between the three definitions. The concordance coefficient was higher for the definitions proposed by Cook and cols. (20) and De Ferranti and cols. (21) and lower among the definitions recommended by the IDF (19) and De Ferranti and cols. (21) in the total sample. When stratified by sex, agreement ranged from mild to moderate. When considering the male gender, the lowest coefficient of agreement was observed between the definitions proposed by the IDF (19) and De Ferranti and cols. (21). For females, the lowest coefficient of agreement was observed between the definitions proposed by Cook and cols. (20) and IDF (19).

**Table 5:** Agreement between the different definitions of Metabolic Syndrome in adolescents aged between 18 and 19 years of the RPS Birth Cohorts. São Luís, 2016

	Criteria	IDF (2007) Coef. (CI 95%)	Cook and cols. (2003) Coef. (CI 95%)	De Ferranti and cols. (2004) Coef. (CI 95%)
Total n = 2,064	IDF (2007)	1	0.48 (0.39-0.57)	0.40 (0.34-0.47)
	Cook and cols. (2003)	0.48 (0.39-0.57)	1	0.56 (0.50-0.63)
	De Ferranti and cols. (2004)	0.40 (0.34-0.47)	0.56 (0.50-0.63)	1
Boys n = 1,015	IDF (2007)	1	0.57 (0.45-0.68)	0.32 (0.23-0.41)
	Cook and cols. (2003)	0.57 (0.45-0.68)	1	0.59 (0.51-0.67)
	De Ferranti and cols. (2004)	0.32 (0.23-0.41)	0.59 (0.51-0.67)	1
Girls n = 1,049	IDF (2007)	1	0.39 (0.26-0.51)	0.51 (0.41-0.61)
	Cook and cols. (2003)	0.39 (0.26-0.51)	1	0.52 (0.41-0.62)
	De Ferranti and cols. (2004)	0.51 (0.41-0.61)	0.52 (0.41-0.62)	1

IDF: International Diabetes Federation; Coef.: Cohen’s Kappa concordance coefficient; 95% CI: 95% confidence interval.

## DISCUSSION

Different definitions of MS have been proposed so far. Therefore, prevalence estimates may vary substantially between populations, depending not only on their characteristics but especially on the diagnostic criteria applied (3). This study showed a statistically significant difference between the estimates of the prevalence of MS in adolescents aged between 18 and 19.

The main problem in diagnosing MS is the unavailability of an accepted global definition of this phenomenon and different cut-off values for each component (2). Other studies evaluating the definitions of MS in adolescents reported variations from 2.7% to 3.8% (6) and from 0.3 to 26.4% (23). For example, in Brazil, ERICA (Study of Cardiovascular Risks in Adolescents) found a prevalence of 2.6% (95% CI: 2.3-2.9) in 37,504 adolescents aged between 12 and 17 using the definition proposed by the IDF (24).

Although most definitions agree on using the same five components, they differ in cut-off points, so individual SM components are weighted differently (23). In this context, the IDF (19) recommends using fixed values as cut-off points like those used in adults. Meanwhile, Cook and cols. (20) and De Ferranti and cols. (21) recommend the assessment based on percentiles adjusted for age and sex, which reflects significant differences between definitions.

Despite its suitability, one of the problems with using percentiles for age and sex in the criteria assessment for MS is the adjustment of the cut-off value in the transition to adulthood since the criteria are not based on percentile distribution in adults but rather fixed values (7). In this context, individuals aged between 18 and 19, as is the case of the sample, can be classified in different ways.

We analyzed the differences between the sexes due to their different hormonal influences during adolescence (25). The prevalence of MS in boys ranged from 3.7% (95% CI: 2.6-5.1) to 13.8% (95% CI: 11.7-16.1). In girls, this variation ranged from 2.8% (95% CI: 1.9-4.0) to 8.8% (95% CI: 7.2-10.7). The ERICA (24) study found the prevalence of MS, according to the IDF (19) criteria, of 2.1% (95% CI: 1.5-2.7) for girls and 3.3% (95% CI: 2.5-4.2) for boys, among Brazilian adolescents aged 15 to 17 years. In Gurka and cols. (26), the authors identified that boys were generally more likely to be diagnosed with MS, as proposed by Cook and cols. (20) in any racial/ethnic group in 4,413 12- to 19-year-olds in the United States.

The possible mechanism underlying the difference between the sexes in the prevalence of MS is still uncertain (27). It is related to different aspects of lifestyle, genetic factors, and racial differences (28). When we specifically analyzed each MS component by sex, according to the definition proposed by the IDF (19), there was a difference between the sexes for all MS components except blood glucose. However, when evaluated according to the definitions proposed by Cook and cols. (20) and De Ferranti and cols. (21), these differences were identified only for TG and HDL, which suggests that the differences found depend on the definition used. Thus, inferences related to sex become limited.

Literature reports that obesity is the most frequent cause of secondary dyslipidemia in adolescence, consisting of an increase in TG and a decrease in HDL (18). In addition, other causes of dyslipidemia in adolescence are related to lifestyle habits, medications, genetic causes, and comorbidities (17).

Despite the different prevalences of MS observed, we found a moderate agreement between the three definitions evaluated, in which the highest coefficients of agreement were observed between the definitions by Cook and cols. (20) and De Ferranti and cols. (21). We believe they are related to the fact that both consider the presence of at least three MS components. These components differ from the requirement of the definition proposed by the IDF (19), in which the presence of altered WC is mandatory. Our results are consistent with previous studies (6-29), which evaluated the agreement between the different definitions of MS in adolescents.

Currently, there is no standard definition for MS. This leads to under or overestimation of its prevalence, severely limiting the comparability of different studies and compromising its usefulness in the clinical setting (30). The increased burden of metabolic alterations in adulthood (8,9,31) has already been reported, reflecting the importance of precociously investigating the topic.

It is important to highlight that our study has some limitations. First, the sample evaluated comprises adolescents aged between 18 and 19, not including the other age groups inherent to adolescence. Therefore, we recommend caution in extrapolating our findings. Due to this age restriction, we assessed only the MS diagnostic criteria encompassing it. Furthermore, it was not possible to assess insulin resistance parameters, as this variable was not evaluated in the study.

Another important aspect is that the collection of biochemical tests evaluated in our study was not performed in a fasting condition. However, the assessment of non-fasting serum glucose levels has the potential to be incorporated into clinical practice as an initial screening and to determine which individuals should undergo definitive tests (16). Regarding the lipid profile, the Update of the Brazilian Guideline on Dyslipidemias and Prevention of Atherosclerosis (17) recommends that fasting is not necessary to perform CT and HDL. It is because the postprandial state does not interfere with the concentration of these particles, and increased postprandial TG levels represent a higher risk for cardiovascular events.

It should be noted that this study has an expressive sample size, which minimizes the occurrence of random error and reinforces the reliability of our analyses.

There is a wide range of criteria for the definition of MS disseminated in the scientific environment, which impacts the diagnosis of MS and comparability between studies. In our study, the MS prevalence more than doubled from the results obtained by three different definitions. Furthermore, even though they showed moderate agreement in the total sample, the differences between the sexes varied according to the definition used.

We did not identify Brazilian studies comparing different definitions for the MS assessment using a sample size like ours. Thus, this study elucidates the difficulty in evaluating MS in this group and aims to stimulate the scientific community to identify ways to better assess metabolic syndrome in adolescents. However, the wide variety of available criteria limits its application since different criteria have different prevalence’s.

It is our understanding that there are distinctions regarding age, sex, and ethnicity in studies involving MS. However, MS can have important repercussions for the health of individuals in their adult phase. Thus, the scientific community needs to identify consensual ways of evaluating the syndrome. Therefore, more studies are needed to investigate this issue in the Brazilian adolescent population and define a consensus on the best way to assess MS in this age group.
